# Editorial: Calcium Metabolism: Hormonal Crosstalk, Pathophysiology and Disease

**DOI:** 10.3389/fmed.2022.899416

**Published:** 2022-04-25

**Authors:** Giulia Battafarano, Grazia Chiellini, Federica Saponaro

**Affiliations:** ^1^Bone Physiopathology Research Unit, Genetics and Rare Diseases Research Division, Bambino Gesù Children's Hospital, IRCCS, Rome, Italy; ^2^Department of Surgical, Medical and Molecular Pathology and Critical Care Medicine, University of Pisa, Pisa, Italy

**Keywords:** calcium homeostasis, calcium metabolism, bone diseases, calcium disorders, mineral homeostasis, PTH, FGF23

Calcium is involved in several biological functions, such as cell signaling, neural transmission, muscle function, blood coagulation, membrane and cytoskeletal regulation, secretion and biomineralisation (1). Its levels are finely regulated in intracellular and extracellular compartments in order to be maintained in a narrow range.

In the intracellular compartment, the regulation of calcium levels is attributed to calcium uptake by mitochondria in order to preserve cellular metabolic homeostasis. Calcium uptake is mediated by the highly selective calcium channel MCU localized on the inner mitochondrial membrane. MCU roles in cell metabolism and cell death are deeply described by the precious review published by Wang et al.

In the body, 99% of the total calcium resides in the skeleton stored as hydroxyapatite and released in serum when it is needed; whereas the extraskeletal component accounts for only 1% (2). Half of calcium introduced by diet is absorbed by the gastrointestinal tract in a vitamin D-dependent manner; it is filtered by renal glomeruli and up to 99% is reabsorbed by passive and active processes. Only 50–250 mg/day is excreted in urine (2, 3). Regulation of calcium tubular reabsorption and urinary excretion contribute to the maintenance of calcemia.

Calcium metabolism is guaranteed by three main players: PTH (ParaThyroid Hormone), Vitamin D, and FGF23 (Fibroblast Growth Factor 23). They synergistically act in a complex hormonal crosstalk within the parathyroid-intestine-bone-kidney axis.

Serum calcium levels are detected by the Calcium-Sensing Receptor (CaSR) highly expressed on cell membrane, able to respond to altered extracellular ionized calcium concentration. In parathyroid glands, its activation induces a signal transduction leading to PTH synthesis suppression. Thus, when serum calcium levels decrease, the CaSR remains inactivated without suppressing the PTH synthesis and secretion. In kidneys, the activation of the receptor reduces the calcium reabsorption (4). The importance of CaSR has been highlighted by disorders associated to its mutations. CaSR heterozygous loss-of-function mutations results in a “desensitization” of the receptor to calcium levels. This makes calcium-sensing stimulation at higher serum calcium levels than in normal conditions, leading to hypercalcemia and hypocalciuria, known as Familial Hypocalciuric Hypercalcemia (FHH), shearing mineral and PTH profile with primary hyperparathyroidism (PHPT) (5). On the contrary, heterozygous activating mutations in the CaSR lead to increased sensitivity of the receptor to extracellular calcium levels, thus inducing the reverse effect in the Autosomal Dominant Hypocalcemia (ADH) (6). Homozygous and heterozygous loss-of-function mutation have been also identified in neonatal severe primary hyperparathyroidism (NSHPT) (7). In this context, Palmieri et al. highlighted the importance to identify the genotype/phenotype correlation for each patient in order to address the correct therapeutic approach on heterozygous hypercalcemic patients with loss-of-function mutation of CaSR who do not benefit from medical or surgical management.

PTH is released from cells tonically and in a pulsatile fashion (8). This hormone acts directly on the kidney and bone *via* PTH1R, a PTH receptor expressed on the surface of tubular cells, osteoblast and osteocytes, and indirectly on gastrointestinal tract (9, 10). On kidney, it is able to induce the increase of calcium reabsorption and the secretion of Vitamin D that, in turn, increases the gut absorption of calcium. On bone, PTH regulates the remodeling inducing directly osteogenesis and bone formation and indirectly osteoclast resorption (11). The final effect on bone mass, either catabolic or anabolic, will depend on the dose and periodicity of the PTH signal (8, 12, 13). When the release of PTH is impaired, a pathological condition as hyperparathyroidism or hypoparathyroidism occurs; each characterized by hypercalcemia or hypocalcemia, respectively. Recent evidences support that the resulting alteration in skeletal muscle are not only due to the altered calcemia but also to direct effect of PTH on that tissue, as reported by Romagnoli and Brandi. In their elegant review, the authors revealed the direct effects of PTH on myocytes function, myotubes formation and modulation of muscle vitamin D uptake and its involvement in muscle regeneration (Romagnoli and Brandi).

The complexity of the system regulating calcium homeostasis is highlighted by the observation reported by Nicoli et al., who, in the description of a population affected by osteopenia/osteoporosis, hyperparathyroidism and hypercalciuria, identified 3 clusters with different mineral metabolic profiles. This observation underlines on one hand how the system of calcium homeostasis is regulated by several inter-connected factors, and on the other hand, that it determines a diagnostic challenge in the distinguishing primary from secondary hyperparathyroidism (Nicoli et al.).

Vitamin D is a fat-soluble hormone that plays a central role in the regulation of mineral homeostasis and skeletal health; it modulates intestinal absorption of calcium and phosphate, renal calcium reabsorption and bone remodeling coordinating with parathyroid functions. The biologically active form is 1,25 dihydroxy-vitamin D synthesized by the skin through sunlight exposure and by metabolic conversion of its precursors vitamin D2 and vitamin D3 introduced by the diet, transported to the liver and then to the kidney where they are hydroxylated to obtain the active form (14–16). Renal synthesis and catabolism are tightly coordinated with the calcium/PTH axis and the phosphate/FGF23 axis. Several extra-skeletal roles of vitamin D have been identified including vascular regulation. Specifically, Bellone et al. for the first time analyzed the interconnection between bisphosphonate related osteonecrosis of the jaw, VEGF (Vascular-Endothelial Growth Factor) and vitamin D levels in postmenopausal osteoporotic women. Even though the study needs to be confirmed by further larger studies, the authors identify a vitamin D-dependent modulation of circulating VEGF (Bellone et al.). Finally, FGF23 is a growth factor produced in bone tissue which hypophosphaturic function is strictly related to PTH and vitamin D.

In conclusion, expanding the knowledge about the interaction between the various hormones involved, collecting new data regarding the interruption of this balance, as well as understanding the molecular pathways involved, is of fundamental importance for a correct and prompt diagnosis and treatment of patients affected by endocrine disorders. All articles published in this special issue provide a significant contribution to the ongoing understanding of calcium metabolisms and hormonal homeostasis. It is a pleasure for the Guest Editor to gratefully acknowledge all the authors for their important and accurate contributions.

## Author Contributions

GB, GC, and FS contributed to conception and design of the editorial. GB wrote the first draft of the manuscript. All authors contributed to manuscript revision, read, and approved the submitted version.

## Conflict of Interest

The authors declare that the research was conducted in the absence of any commercial or financial relationships that could be construed as a potential conflict of interest.

## Publisher's Note

All claims expressed in this article are solely those of the authors and do not necessarily represent those of their affiliated organizations, or those of the publisher, the editors and the reviewers. Any product that may be evaluated in this article, or claim that may be made by its manufacturer, is not guaranteed or endorsed by the publisher.

## References

[1] Yu E Sharma S. Physiology, Calcium. Treasure Island: StatPearls Books (2021).

[2] Moe S. M. Calcium homeostasis in health and in kidney disease. Compr Physiol. (2016) 15:6. 10.1002/cphy.c150052 27783859

[3] Heaney RP Skillman TG. Secretion and excretion of calcium by the human gastrointestinal tract. J Lab Clin Med. (1964) 1964:64. 14192566

[4] Papadopoulou A Bountouvi E Karachaliou FE. The molecular basis of calcium and phosphorus inherited metabolic disorders. Genes. (2021) 12:5. 10.3390/genes12050734 34068220 PMC8153134

[5] Christensen SE Nissen PH Vestergaard P Heickendorff L Rejnmark L Brixen K. Plasma 25-hydroxyvitamin D, 1,25-dihydroxyvitamin D, and parathyroid hormone in familial hypocalciuric hypercalcemia and primary hyperparathyroidism. Eur J Endocrinol. (2008) 159:6. 10.1530/EJE-08-0440 18787045

[6] Pearce SH Williamson C Kifor O Bai M Coulthard MG Davies M. A familial syndrome of hypocalcemia with hypercalciuria due to mutations in the calcium-sensing receptor. N Engl J Med. (1996) 335:15. 10.1056/NEJM199610103351505 8813042

[7] Gorvin.CM. Molecular and clinical insights from studies of calcium-sensing receptor mutations. J Mol Endocrinol. (2019) 63:2 10.1530/JME-19-0104 31189130

[8] Silva BC Bilezikian JP. Parathyroid hormone: anabolic and catabolic actions on the skeleton. Curr Opin Pharmacol. (2015) 22:810–1. 10.1016/j.coph.2015.03.005 25854704 PMC5407089

[9] Datta NS Abou-Samra AB. PTH and PTHrP signaling in osteoblasts. Cell Signal. (2009) 21. 10.1016/j.cellsig.2009.02.012 19249350 PMC2723940

[10] Fermor B Skerry TM. PTH/PTHrP receptor expression on osteoblasts and osteocytes but not resorbing bone surfaces in growing rats. J Bone Miner Res. (1995) 10:12. 10.1002/jbmr.5650101213 8619374

[11] Kousteni S Bilezikian JP. The cell biology of parathyroid hormone in osteoblasts. Curr Osteoporos Rep. (2008) 6:2. 10.1007/s11914-008-0013-9 18778567

[12] Onyia JE Helvering LM Gelbert L Wei T Huang S Chen P. Molecular profile of catabolic versus anabolic treatment regimens of parathyroid hormone (PTH) in rat bone: an analysis by DNA microarray. J Cell Biochem. (2005) 95:2. 10.1002/jcb.20438 15779007

[13] Locklin RM Khosla S Turner RT Riggs BL. Mediators of the biphasic responses of bone to intermittent and continuously administered parathyroid hormone. J Cell Biochem. (2003) 89:1. 10.1002/jcb.10490 12682918

[14] Jean G Souberbielle JC Chazot C. Vitamin D in chronic kidney disease and dialysis patients. Nutrients. (2017) 9:4. 10.3390/nu9040328 28346348 PMC5409667

[15] Parfitt AM Gallagher JC Heaney RP Johnston CC Neer R Whedon GD. Vitamin D and bone health in the elderly. Am J Clin Nutr 36:5 Suppl. (1982). 10.1093/ajcn/36.5.1014 6765074

[16] Jones G Prosser DE Kaufmann M. Cytochrome P450-mediated metabolism of vitamin D. J Lipid Res. (2014) 55:1. 10.1194/jlr.R031534 23564710 PMC3927478

